# Fog Density Analysis Based on the Alignment of an Airport Video and Visibility Data

**DOI:** 10.3390/s24185930

**Published:** 2024-09-12

**Authors:** Mingrui Dai, Guohua Li, Weifeng Shi

**Affiliations:** Institute of Computing Technology, China Academy of Railway Sciences Co., Ltd., Beijing 100081, China; daimr@rails.cn (M.D.); guohua.li@rails.cn (G.L.)

**Keywords:** fog density estimation, visibility, alignment, digital video streams, digital optical images, fog density variation model, fog density change prediction, airport fog, foggy image, foggy video

## Abstract

The density of fog is directly related to visibility and is one of the decision-making criteria for airport flight management and highway traffic management. Estimating fog density based on images and videos has been a popular research topic in recent years. However, the fog density estimated results based on images should be further evaluated and analyzed by combining weather information from other sensors. The data obtained by different sensors often need to be aligned in terms of time because of the difference in acquisition methods. In this paper, we propose a video and a visibility data alignment method based on temporal consistency for data alignment. After data alignment, the fog density estimation results based on images and videos can be analyzed, and the incorrect estimation results can be efficiently detected and corrected. The experimental results show that the new method effectively combines videos and visibility for fog density estimation.

## 1. Introduction

As a common meteorological phenomenon, fog density is closely related to climate and weather patterns. In daily life, foggy weather significantly impacts on traffic safety management, especially in scenarios such as airports and expressways. Real-time monitoring and analysis of fog density can provide decision-making support for traffic management departments, ensure timely safety measures are taken, and reduce the occurrence of traffic accidents. For example, when the fog density is too high, stopping flights from landing and taking off and closing highways are common strategies; when the fog density is high, traffic management departments often limit the number of takeoffs and landings of flights and implement flow restrictions on highways.

Traditionally, fog density estimation relies on specialized measuring equipment to gather data. This method boasts high accuracy and strong real-time performance, making it a reliable choice. However, the downside is the relatively high costs associated with purchasing, installing, using, and maintaining such equipment, as well as their bulkiness, which can hinder portability.

In recent years, with the widespread use of photography and video equipment, researchers have mainly focused on the methods for estimating fog density based on images and videos. The advantage of this method is that compared to professional measuring equipment, the cost of the camera is lower; it is capable of conducting non-contact measurements, which has significant advantages in specific hazardous or inaccessible environments; repeated measurements, multi-person measurements, and verification can be performed if these foggy images have been saved. Of course, from the currently published results, the disadvantage of this method is that its accuracy is not high, and the validation of various methods is insufficient.

In this paper, we propose a method for analyzing fog density in video images based on monitoring videos and visibility data obtained from sensors. A prerequisite for this method to achieve good performance is the alignment, in terms of time, of monitoring video data with visibility data obtained by sensors. Because the video data are continuously captured over time, while the visibility data obtained by sensors only have several time nodes per minute, which is discrete, the first challenging task of this paper is to align these two data according to time nodes. In addition, these data are captured in a real-time monitoring scenario, often in the wild, so there are significant changes in lighting conditions and camera shaking, which increases the difficulty of data analysis and processing. The main highlight of this paper is the proposal to use visibility data to evaluate a video’s fog density estimation results. In order to achieve this goal, we construct three algorithms that address the time information identification, the data alignment, and the fog density evaluation.

**Figure 1:**
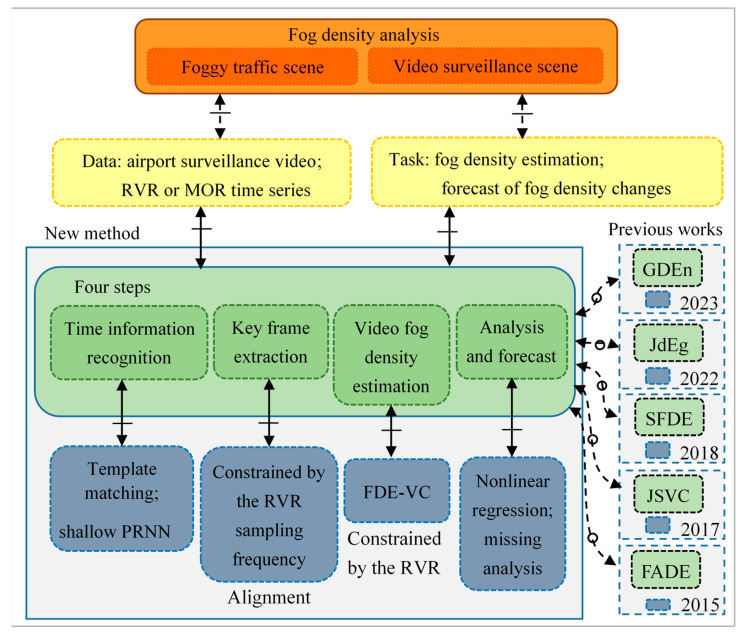
The objective and main content of this paper are presented through the NBGM.

The goal, data, tasks, proposed algorithms, and previous works have been visualized using a nested blocks and guidelines model (NBGM) [1], a data visualization method shown in Figure 1.

## 2. Related Works

This paper focuses on the fog density analysis of video. Our method is related to the time alignment of the video and the visibility data. Therefore, the related work is now surveyed from these two aspects.

### 2.1. Image Fog Density Analysis

The fog density analysis of images and videos includes fog density estimation and visibility analysis. Methods for estimating fog density through images according to the object can be roughly classified into two categories: fog density measured as discrete levels and fog density measured as a continuum. In addition, the estimation of fog density involves the construction of datasets and the evaluation of estimation methods. Below, we discuss the current state of research on each topic.

The first category is to estimate the fog density level of a foggy image. According to the fog density level, images can be classified into two categories, fog-free and fog, or four categories: fog-free, thin, medium, and heavy fog. This problem is related to cluster and classification. Therefore, the machine learning method can be employed. In 2018, M. I. Anwar et al. [2] presented a method for estimating the fog density level using a Support Vector Machine (SVM), which plays a key role in classifying the synthetic data into two classes, homogeneous fog and heterogeneous fog. In 2019, Y. Chen et al. [3] proposed a fog density estimation algorithm using multiple features, including color, edge gradient, and transmittance. Their classification is completed by training an SVM classifier. Their experiments are conducted based on three levels of image data: fog-free, thin fog, and dense fog images. In 2020, J. Dong et al. [4] built a model based on four features: color, dark channel, image entropy, and contrast. They used a multi-classification algorithm, S-DAGSVM, to classify four fog density levels: fog-free, light, medium, and dense fog. In 2023, W. Yang et al. [5] proposed a deep learning framework named VENet based on multiple visual feature fusion for fog visibility estimation. Their method comprises two subtask networks for fog level classification and fog visibility estimation, respectively. They employed a special feature extractor and an anchor-based regression method (ARM) to improve the accuracy. Five fog levels, fog-free, low, medium, high, and dense fog, were classified in their experiments.

Besides fog level evaluation using machine learning methods, some researchers devoted themselves to adopting methods based on physics, mechanics, mathematics, and statistics to avoid black box trouble from deep learning and lack of high-quality training data. Applying image entropy for fog density analysis and constructing dehazing algorithms [6] is practicable. For example, in 2023, R. Cao et al. [7] proposed an image-based method to estimate fog density levels to improve the accuracy and efficiency of analyzing meteorological conditions and validating fog density predictions. Their method used two types of image entropy: a two-dimensional directional entropy derived from four-direction Sobel operators and a combined entropy that integrates the image directional entropy and grayscale entropy. We name this method as GDEn.

The second category estimates the fog density of an image, and the value range of fog density is non-negative continuous real numbers. The current primary method is still based on mechanism modeling for this continuous estimation problem, which is not a simple classification. The main idea of this category of method is to establish a mapping function or model between the image fog density and the visibility or image features. For example, a referenceless perceptual fog density prediction model, called Fog Aware Density Evaluator (FADE), based on natural scene statistics (NSS) and fog-aware statistical features, was proposed by L. K. Choi in 2015 [8]. FADE predicts the visibility of a foggy scene from a single image without reference to a corresponding fog-free image, without dependence on salient objects in a scene, without side geographical camera information, without estimating a depth-dependent transmission map, and without training on human-rated judgments. FADE only uses measurable deviations from statistical regularities observed in natural foggy and fog-free images. In 2018, Z. Ling et al. [9] developed a simple fog density evaluator (SFDE) by adopting a linear combination of three fog-relevant statistical features: chroma variance, average saturation, and Weber luminance contrast. These three features were selected by analyzing thirteen features of the image. In order to estimate fog density correctly and to remove fog from foggy images appropriately, a surrogate model for optical depth was presented by Y. Jiang et al. in 2017 [10]. We name this model JSVC because it consists of three image features: dark channel proposed by K. He [11], the saturation value of an image in HSV format, and chroma in the CIELab color space.

For the convenience of expression, researchers usually construct an index representing fog density; for example, the fog density index had been defined as a function of the dark channel information and the pseudo-edge details information of the images [12], named JdEg. As an application, this metric (JdEg) can be used to evaluate the effectiveness of dehazing algorithms. However, most of these existing methods did not use visibility information and did not analyze the abnormal video frames.

### 2.2. The Alignment of Video-Related Data

The alignment of video-related data is an essential foundation for video analysis. Early in 1995, Bajura et al. [13] pointed out that computer-generated objects must be visually registered concerning real-world objects in every image in video-based augmented reality systems. In recent years, multiple sensor devices have been used more comprehensively to obtain data from the same scene. The data collected by different sensor devices need to be calibrated and aligned synchronously in time. Wang L. et al. [14] addressed the problem of multi-sensor data fusion and time alignment in the case of considerable data transmission delay. Their algorithm aligned the sampling time through the Kalman filter alignment algorithm and used the weighted fusion to get the fusion estimation at the sampling time. In 2021, Kyrollos D. G. et al. [15] collected a multi-modal neonatal patient dataset, which was simultaneously collected from an RGB-D video camera placed above the patient and a pressure-sensitive mat (PSM) beneath the patient. They explored using various transforms to achieve alignment between the video image plane and the PSM. In 2003, Rao C. et al. [16] proposed a method to establish temporal correspondence between the frames of two videos. This method uses a temporal pyramid of trajectories to improve the accuracy of their view-invariant dynamic time-warping approach. This way, videos of individuals taken at different times and from distinct viewpoints can be synchronized. In 2015, Bojanowski P. et al. [17] addressed modal data, a set of videos along with natural language descriptions in the form of multiple sentences, and that these sentences appear in the same temporal order as their visual counterparts. They proposed a method for aligning the two modalities, i.e., automatically providing a time stamp for every sentence. They proposed to cast this task as a temporal assignment problem with an implicit linear mapping between the two feature modalities. They formulated this problem as an integer quadratic program and solved its continuous convex relaxation using an efficient conditional gradient algorithm. In 2016, Dogan P. et al. [18] proposed a method for temporally aligning the video frames with the sentences using both visual and textual information, which provides automatic timestamps for each narrative sentence. They computed the similarity between both types of information using vectorial descriptors and proposed to cast this alignment task as a matching problem that we solve via dynamic programming.

From another perspective, video is also a time series problem to some extent, for example, the alignment of two time series [19]. It is no doubt that alignment based on curve fitting is effective for time series composed of numbers [20].

However, the existing published methods described above have largely overlooked the alignment between videos and visibility sensing data. There is a scarcity of articles that evaluate the results of fog density estimation based on video using visibility information. Our research, focusing on the alignment of multi-modal remote sensing data, aims to find a solution. The images and videos captured in natural environments, due to changes in lighting and weather [21], introduce a significant amount of noise, thereby posing substantial challenges for data alignment.

## 3. Data and the Proposed Methods

The proposed method and the data we used to evaluate our method are described in this section.

### 3.1. Data

**Figure 2:**
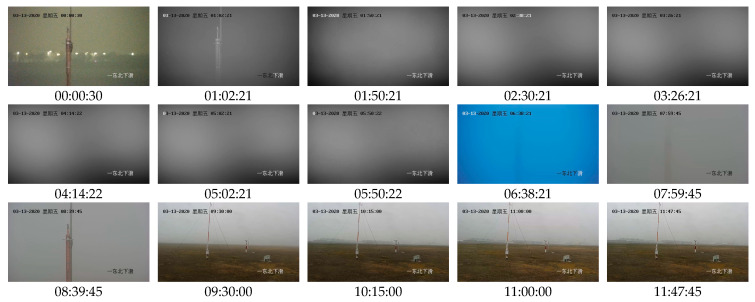
Fifteen keyframes are sampled from an airport monitoring video. The time interval between adjacent keyframes is about 40–82 min. The number below each subgraph represents the monitoring time for capturing this frame. Note: The information in the upper left corner of each frame image is the date (13 March 2020), Friday, and time; the information in the bottom right corner of each frame image is the orientation, i.e., Northeast Decline.

The data are from the fourth question in the 2020 China Post-Graduate Mathematical Contest in Modeling (https://cpipc.acge.org.cn/cw/hp/4, accessed on 12 March 2024) and can be downloaded from the website. Among these data, a video and a visibility Excel file are used in our experiments in this paper. The video was captured by a fixed-point rotatable camera. This video records an airport scene from 00:00:26 to 11:47:48 on March 13 (Friday), 2020. This morning is foggy. The size of each frame is 1280×720. The default play speed of the video is 25 frames per second. Figure 2 displays fifteen key frames of the video. The time intervals of these fifteen frames range from 40 to 82 s for displaying some remarkable changes in frames, such as lighting and color. From these subfigures, it can be found that the light, the text color, and the fog density are various.

**Figure 3:**
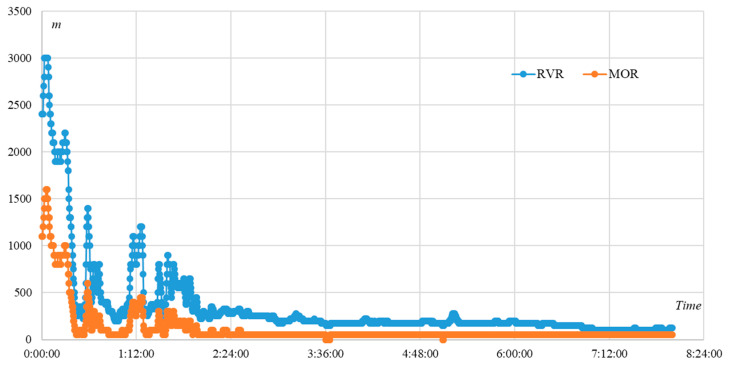
The changes in visibility, represented by RVR and MOR, at this airport from 0:00:00 to 7:59:45.

Accompanying this video, there is an Excel file that records the visibility, represented by Runway Visual Range (RVR) and Meteorological Optical Range (MOR), at this airport from 12 March 2020 8:00:00 to 13 March 2020 7:59:45. Four measurements are recorded at equal intervals every minute, resulting in four recorded visibility values per minute. The number of data points is 1918, as shown in Figure 3. Several statistical information, including maximum value, minimum value, average, and standard deviation, about the visibility is listed in Table 1.

By comparing the video to the visibility data, it can be easily found that the time overlap interval between these two is from 0:00:30 to 7:59:45; two series of data are with different sample time frequencies.

### 3.2. The Proposed Method

The airport’s video surveillance and visibility measurement data can be used to monitor, analyze, and predict changes in fog density. To achieve this task, we propose a video Fog Density Estimation algorithm based on Visibility Calibration, named FDE-VC, and use it as a basis for fog density analysis and prediction, providing decision-making reference for airport management. The proposed method consists of four steps: image-based time information recognition, time-constrained key frame extraction, FDE-VC algorithm, and forecast of fog density changes.

#### 3.2.1. Image-Based Time Information Recognition

**Figure 4:**
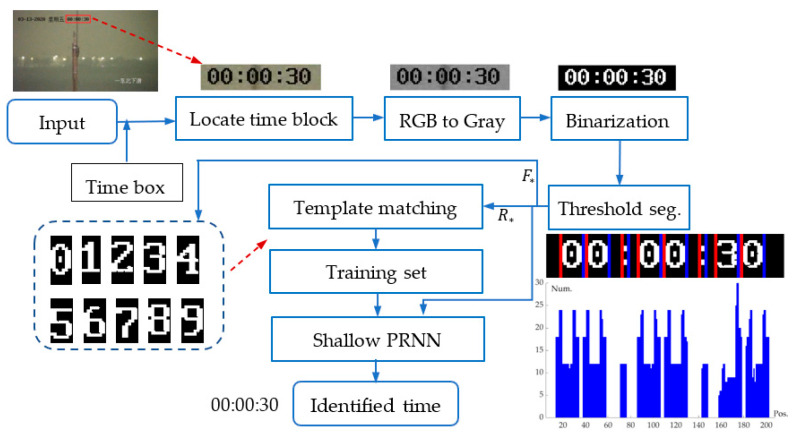
The flowchart of the ERTimeInfo algorithm. $F_{*}\;$represents that ten figure templates are obtained. $R_{*}$ represents that 500 random frames of the video are extracted for constructing the training set. Note: The information in the upper left corner of the input frame image is the date (13 March 2020), Friday, and time (00:00:30); the information in the bottom right corner of each frame image is the orientation, i.e., Northeast Decline.

Video-based fog density estimation and analysis are time-dependent. Therefore, we need first to identify the temporal information in the video. Figure 2 shows that the temporal information is displayed in the top-left corner of each frame. By specifying a fixed box, the position of time information can be located and partitioned. We use threshold segmentation to get figure blocks. Then, the figure template matching algorithm is employed to recognize time digits from the 500 randomly sampled frames. After manual checking, we use these identified figures to form a training set. Finally, we use a shallow neural network for pattern recognition (Shallow PRNN) [22] to obtain the time information. The proposed algorithm, Extraction and Recognition of Time Information (ERTimeInfo) algorithm, is illustrated in Figure 4.

The ERTimeInfo algorithm consists of nine steps: (S1) input image and time box position, (S2) locate time block, (S3) convert the RGB image to the gray image, (S4) binarization, (S5) threshold segmentation, (S6) build the figure template, (S7) roughly identify figures using template matching method which is recommended in various detection task [23], (S8) fine identify figures using the Shallow PRNN, (S9) output the identified time information. Each step, except for S1 and S9, will be detailed below.

S2: This locating time block step is implemented by specifying the position of the time box’s boundary in the frame image.

S3: The step of converting the RGB image to the gray image is completed using the rgb2gray function from Matlab. This function is constructed by formula $G\left(x\right)=0.2989R(x)+0.5870G(x)+0.114B(x)$, where *x* is any a pixel in the image and R, G, and B are the color values of three channels, red, green, and blue, respectively.

S4: In Figure 2, it can be found that time numbers in the video are sometimes white and sometimes black. So, our binarization formula can be defined as:

(1) $$b\left(x\right)=\left\{\begin{array}{c} 1,\;\;x<\sigma_{0}\;or\;x>\sigma_{1}\\ 0,\;\;otherwise\;\;\;\;\;\; \end{array},\right.$$

where x is the grayscale value of a pixel and $\sigma_{0}$ and $\sigma_{1}$ are parameters specified by the user. In our experiment, the default values for these two parameters are $\sigma_{0}=0.2$ and $\sigma_{1}=0.9$.

S5: In the threshold segmentation step, we calculated the number of white pixels (Num.) in each column along this binarization image block from left to right, and the frequency statistics are illustrated in the subgraph in the most right of Figure 4. In this way, when Num.>0, this subblock, using the red to blue lines to indicate the boundary, has a figure or a colon.

S6: The figure template is built after the threshold segmentation step. The ten figure templates are built using the partitioned figures of frames randomly sampled from the first ten seconds of the video.

S7: In the step of roughly identifying figures using the template matching method, we randomly sample 500 video frames. Then, we use the template matching method to identify those figures in these frames. At first, the test block containing a figure is resized to match that of the figure template before identification. The matching method is defined by the maximum correlation coefficient between the block and the template as the best matching degree to identify these numbers. That is:

(2) $$l^{*}\left(B_{t}\right)=\arg\underset{i=0,1,\cdots ,9}{\max}\rho (B_{t},T_{i}),$$

where $B_{t}$ is the test subblock, $T_{i}(i=0,1,\cdots ,9)$ are the template of figure i, and$\;\rho (B_{t},T_{i})$ is the correlation coefficient between $B_{t}$ and $T_{i}$. These identified figures consist of a training set.

S8: Finally, identify figures using the Shallow PRNN. The reason why we adopt this step is that our experiments have shown that the template matching method has lower accuracy in digit recognition than neural networks, and its robustness is also slightly inferior. With a classical Shallow PRNN already integrated into Matlab 2018, we can easily integrate this module into our algorithm framework, as shown in Figure 4.

#### 3.2.2. Time-Constrained Key Frame Extraction

The visibility data represented by RVR and MOR (Figure 3) are recorded in discrete format, which is recorded four times per minute, with a 15 s interval between adjacent time points. By contrast, the airport surveillance video is recorded in continuous format. When this video is played, it can be discretized into 25 frames per second. However, we cannot extract the key frames using equal spacing, because there are several abnormal cases in the video.

**Figure 5:**
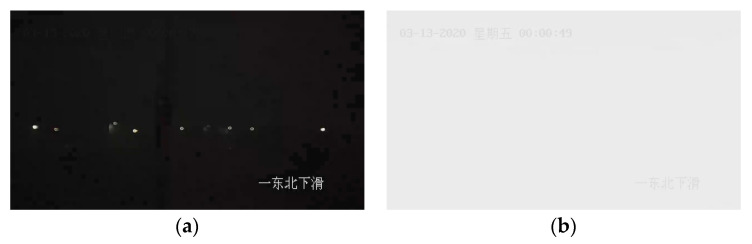
Black screen frame (**a**) and white screen frame (**b**). The black screen occurs in the 18th frame of the video; the white screen frame occurs in the 49th second of the video. Note: The information in the upper left corner of image (**b**) is the date (13 March 2020), Friday, and time (00:00:49); the information in the bottom right corner of each frame image is the orientation, i.e., Northeast Decline.

(1) There are a lot of abnormal frames, including black screen frames (Figure 5a) and white screen frames (Figure 5b).
(2) The recorded time point of this video is intermittent. For example, after 00:47:29, the video jumps to 01:01:29, with about 14 min of video recording missing.

Due to these anomalies in the video, we need to identify the time in the video and align the visibility data with the video according to the time information.

Our alignment method is time-constrained key frame extraction. The key frame is defined by the time from the visibility file. In this way, the extracted key frames are consistent with the time of the visibility data. The solution to several abnormal situations follows the following rules.

(1) For abnormal frames, if there is an exception in a single frame, it can be replaced with the previous or next frame. For consecutive abnormal frames, linear interpolation is performed using normal frames before and after.
(2) For the missing time in the video, key frames during this period are not extracted. The density estimation of fog during this period will be calculated based on visibility using our model introduced in the following subsection, which is one of the differences between our method and existing video-based fog density estimation methods.
(3) Generally speaking, the interval for collecting visibility data is 15 s. If there are missing data during the data collection, the corresponding key frame at that time point will still be extracted.

#### 3.2.3. FDE-VC Algorithm

The difference from existing methods of estimating the image and video fog density is that the estimated fog density using our method is calibrated using visibility information. The proposed method is named the FDE-VC algorithm.

In the data described in Section 3.1, the visibility information is represented by RVR and MOR (Figure 3). By calculating, the correlation coefficient between the sample observation values of RVR and MOR is 0.9870, which means a strong linear correlation between the values of RVR and MOR. Therefore, we only use the RVR data in our model.

Referring to the exponential model [24,25], we use a non-linear map to describe the relationship between the visibility and the fog density as follows:

(3) $$f_{den}=C_{0}e^{\alpha \cdot d_{vis}},$$

where $f_{den}$ designates the fog density at visibility $d_{vis}$ and $C_{0}\;$and α are parameters. To determine these parameters in Equation (3), the aligned data and a non-linear regression analysis can be used. Note that for Equation (3), the natural logarithm can be taken on both sides simultaneously, and then the non-linear regression can be transformed into linear regression. The subsequent calculations are relatively simple, so we will not describe them here. In summary, the estimated fog density can be evaluated and analyzed using the FDE-VC algorithm (Algorithm 1) as follows.
**Algorithm 1:** FDE-VC**Input:** fog video, RVR. **Output:** Adjusted estimated fog density. (1) Align the video and the visibility data using the method described in Section 3.2.2; (2) Estimate the fog density of each frame of the video using any existing method; (3) Smooth the estimated fog density and the visibility data using the moving average method; (4) Fitting the data according to Equation (3); (5) Find the outlier values of the estimated fog density using the moving averages and dynamic thresholds method [26]; (6) Correct the estimated fog density using Equation (3). (7) Output the corrected fog density of the video.

#### 3.2.4. Forecast of Fog Density Changes

After introducing the exponential model (Equation (3)), the relationship between fog density and visibility is defined. Therefore, these two data can be used for prediction, including using another quantity to estimate when one of the data has local missing data and fill in the missing data. For our data, the video has some missing frames, so the fog density at these times cannot be calculated based on the video, but estimated using Equation (3), where the parameters have been estimated in Section 3.2.3.

For fog density forecast, our primary focus is on the trend of fog density changes and the diffusion time of fog, as the diffusion time of fog in the airport directly affects the decision-making behavior of airport management. We adopt a bivariate time series prediction model is used to accomplish this task.

## 4. Experimental Results

We use the data described in Section 2.1 to evaluate our proposed method introduced in Section 3 and analyze and process the airport video and corresponding visibility data. All experiments were conducted on a personal laptop. The laptop’s configuration is an 11th Gen Intel(R) Core(TM) i7-11800H @ 2.30GHz with 64.0 GB RAM. The programming environment is Matlab 2018. The experimental results are as follows.

### 4.1. Results of Identified Figures

Using our ERTimeInfo algorithm described in Section 3.2, the figures from 0 through 9 can be successfully segmented and identified. The segmented accuracy is 100%. After this step, the training set is built, which consists of 509 images labeled as ten classes. There are 52, 35,36, 63, 101, 100, 35, 33, 30, and 24 images, labeled 0, 1, 2, 3, 4, 5, 6, 7, 8, and 9 respectively. A sample confusion matrix using the template matching method is shown in Table 2. From the table, it can be found that only one image’s predicted label is incorrect; it incorrectly identifies 1 as 4, and thus, the identification accuracy of this time is 99.8%. We test the method for time digit recognition of all video frames; the identification accuracy of the template matching method is 94.0%.

For comparison, we tested the Shallow PRNN algorithm on this training set. The parameters are set as follows: the hidden layer size is 17, and the ratio of training, validation, and test are 70%, 15%, and 15%, respectively. The identification accuracies in training, validation, and test sets are all 100%. Using this trained neural network for time digit recognition of all frames of the video, 2773 key frames are output. Unfortunately, seven key frames were not detected. In addition, there are 23 frames of time recognition errors. Therefore, the total accuracy of the Shallow PRNN algorithm is 98.9%.

### 4.2. Key Frame Extraction Results

According to the time points in the visibility file, the key frames have been successfully extracted using our ERTimeInfo algorithm described in Section 3.2. As a result, there are 2773 frames have been extracted; among them, there are 1861 frames that fall into 00:00:30 to 07:59:45. The number of time nodes of visibility data from 00:00:00 to 07:59:45 is 1918. Although the interval time between adjacent key frames and visibility records is both 15 s, there is a significant difference in the number of records. The reason for this difference can be found by aligning these data.

**Figure 6:**
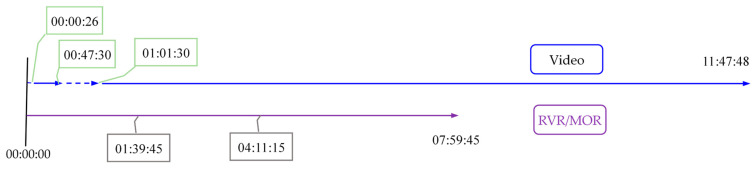
Alignment of the video and the visibility data in the view of time axis. The time in the annotation box indicates missing data.

The result of aligning the video key frames and the time points of recording RVR or MOR is illustrated in Figure 6. We can find that the missing time interval of the video is from 00:00:00 to 00:00:26, and from 00:47:30 to 01:01:30, corresponding to 59 missing key frames; there are two missing visibility records at different time points, i.e., 01:39:45 and 04:11:15. In summary, from 00:00:00 to 07:59:45, there should be 1920 visibility records at different time points and 1920 corresponding key frames, but the actual data obtained showed that the visibility data was missing for two time nodes and key frames were missing for 59 time nodes.

### 4.3. Model Solution

According to Equation (3), we can model the relationship between the estimated fog density and the visibility data using the FDE-VC algorithm. Five fog density estimation models, GDEn [7], FADE [8], SFDE [9], JSVC [10], and JdEg [12], are used for testing the model.

**Figure 7:**
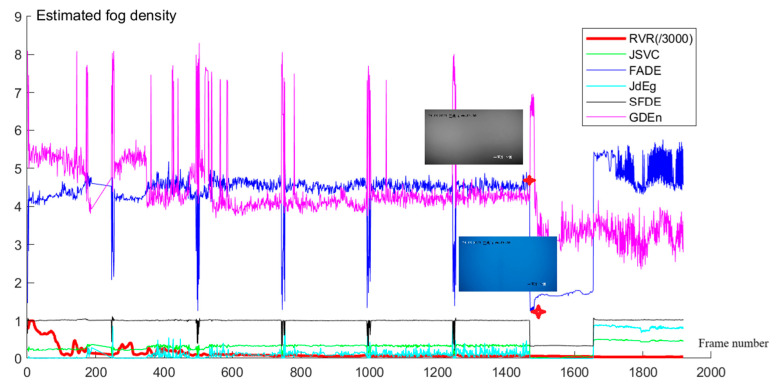
RVR and the fog density values of key frames of the video are estimated using the FADE, JSVC, SFDE, JdEg, and GDEn methods. The time of two key frame images labeled with red stars are 6:07:00 and 6:08:00.

From the aligned data, we use time nodes that includes both video keyframes and visibility measurements. Based on these keyframes, fog density values can be estimated using GDEn, FADE, JSVC, SFDE, and JdEg methods. The estimated results are shown in Figure 7. For convenient comparison, RVR values in the same time nodes are also drawn in the figure and are all divided by 3000. From the figure, it can be found that the GDEn, FADE, and SFDE methods have a more pronounced representation of abnormal frames, as their fog density variation curves fluctuate more violently; from the 188th to the 246th frame, the fog density curves are all straight line segments, because the key frames during this period are missing; although the visibility did not change significantly from frame 1470 to frame 1655, the estimated value of fog suddenly decreased sharply, resulting from a drastic change in color. For this case, the estimated fog density can be calibrated using our method, described in the following subsection.

Using the estimated fog density values and the RVR values and after removing those outliers (for example, the principle of triple standard deviation), Equation (3) can be fitted out. The regression coefficients and several related statistical indicators are listed in Table 3. From the table, it can be found that after fitting, the JdEg method shows the best $R^{2}$, while SFDE method yields the most minor fitting error.

### 4.4. Missing Analysis and Prediction Results

**Figure 8:**
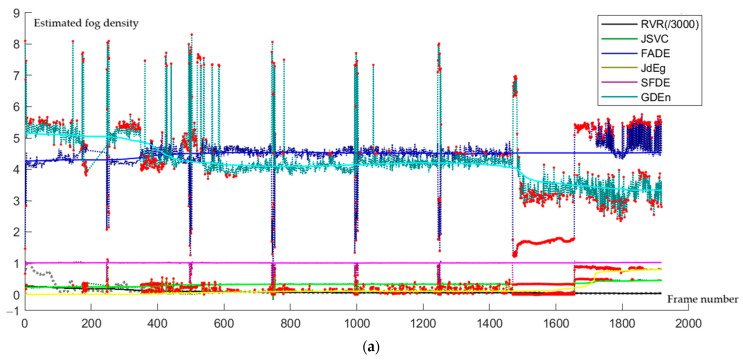

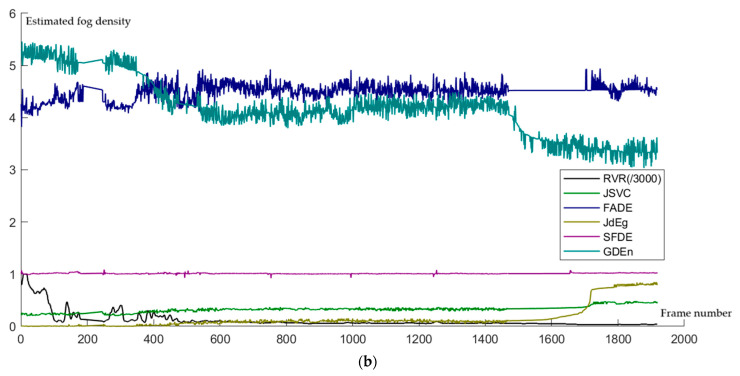
RVR-constrained outlier points are detected, and the smoothing of fog density values is estimated using JSVC, FADE, JdEg, SFDE, and GDEn methods. (**a**) The detected outlier points are labeled in red. (**b**) The detected outlier points have been processed using the movement average approach.

We use the moving averages and dynamic thresholds method [26] to detect the outlier values of estimated fog density. The moving window size is one-sixth of the total frames, and the dynamic threshold is half the standard deviation. In our experiments, outlier values are defined according to this rule: the estimated fog density is an outlier value if it does not belong to this interval [μ−βσ,μ+βσ], where μ is the smooth value at this time node and σ is the standard deviation of the estimated fog density series. β=0.5 is set as the default coefficient in our experiments. According to this outlier detection rule, the outliers detected by the five methods have been plotted as red points in Figure 8a. From this graph, it can be seen that those obvious outliers have been successfully detected. The number of detected outlier points in these five fog density series estimated using JSVC, FADE, JdEg, SFDE, and GDEn methods, are 439, 379, 679, 206, and 508, respectively, which means that among the five estimation results, the volatility of the estimation results from SFDE is the smallest. For those outlier points, the estimated fog density values are corrected using the moving average values, illustrated in Figure 8b.

In the experimental data described in Section 3.1, between 8:00:00 and 11:47:45, there were only video data and no visibility measurement results. Therefore, we estimate the visibility (RVR) values during this period based on the Equation (3) and those estimated fog densities. The Equation (3) can be rewritten as:

(4) $$d_{vis}=\frac{1}{\alpha }\left(\ln f_{den}-\ln C_{0}\right),$$

**Figure 9:**
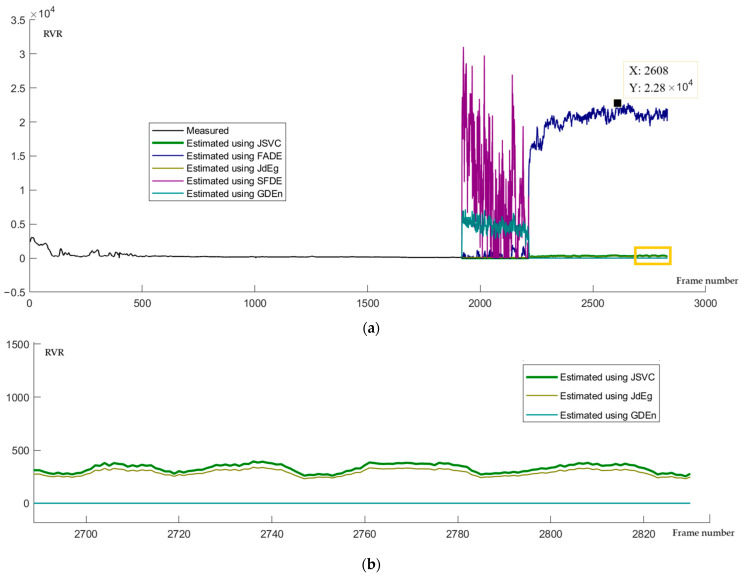
Estimated RVR from 8 a.m. to 12 a.m. (**a**) The RVR value obtained from measurements before 8 a.m. and the visibility value calculated using the Equation (4) based on fog density values estimated by five methods. (**b**) An enlarged view of a time slice labeled with a golden color rectangle in (**a**).

In engineering calculations, one particular case needs to be addressed about Equation (4): when α=0 and $d_{vis}<0$, the value of $d_{vis}$ is specified as zero. The calculated RVR values from 8:00 to 11:47:45 have been drawn as lines in Figure 9a. In this figure, the RVR values are obtained from measurements before 8 a.m. and the visibility values are calculated using Equation (4) based on fog density values estimated by the JSVC, FADE, JdEg, SFDE, and GDEn methods. Figure 9b shows an enlarged view of a time slice (from 11:12:15 to 11:47:45) labeled with a golden color rectangle in Figure 9a. Comparing the forecasted RVR values to the video frames listed in Figure 3, we can find that after 8 o’clock, there has been a significant improvement in visibility. However, there is still moderate fog affecting the visible distance. Therefore, the visibility values estimated by FADE and SFDE are overestimated; the visibility values estimated byGDEn, JSVC, and JdEg are slightly underestimated.

## 5. Conclusions

For the foggy video and the visibility data captured in the same foggy scene, in this paper, we propose a solution that combines these two types of data for mutual verification. The algorithm of data alignment between the video data and the visibility data under the condition of consistent time nodes has been developed in detail. Based on the aligned data, several applications were proposed and demonstrated, including outlier points detection, outlier values correction, the evaluation of the estimated fog density according to the visibility information, and forecasting the visibility based on the estimated fog density.

The experimental results illustrate that the newly proposed method is effective for analyzing the estimated fog density and the visibility data.

From the experimental results, there are a few interesting research findings as well. First, the JdEg method shows the best $R^{2}$ among the five fog density estimating methods, i.e., JSVC, FADE, JdEg, SFDE, and GDEn, according to the map between the visibility and the estimated fog density values. Second, the estimation results of visibility based on the fog density values estimated by these five methods may be overestimated or underestimated.

These findings suggest that a worthwhile direction for future research is to design more accurate fog density estimation methods based on videos and images. Another direction is to study the functional relationship between fog density values and visibility. In addition, in the time identification step, there are some cases of misidentification when the Shallow PRNN algorithm is employed. Typical problems include distinguishing errors between 1 and 4 and 0 and 9, losing seven keyframes, and incorrectly extracting 12 frames in 2773 frames, which affects the efficiency of subsequent processing. Therefore, it is necessary to test more deep learning methods that can handle complex lighting conditions in future work, including ensemble learning methods for multiple deep learning models [27].

## Figures and Tables

**Table 1:** The maximum value (Max.V), minimum value (Min.V), average (Ave.), and standard deviation (Std.) about the visibility of the airport scene.

Index	Max.V	Min.V	Ave.	Std.
RVR	3000	100	345.3597	461.0571
MOR	1600	0	117.7529	217.4216

**Table 2:** The confusion matrix using the template matching method.

Label	0	1	2	3	4	5	6	7	8	9	Sum
0	52	0	0	0	0	0	0	0	0	0	52
1	0	34	0	0	1	0	0	0	0	0	35
2	0	0	36	0	0	0	0	0	0	0	36
3	0	0	0	63	0	0	0	0	0	0	63
4	0	0	0	0	101	0	0	0	0	0	101
5	0	0	0	0	0	100	0	0	0	0	100
6	0	0	0	0	0	0	35	0	0	0	35
7	0	0	0	0	0	0	0	33	0	0	33
8	0	0	0	0	0	0	0	0	30	0	30
9	0	0	0	0	0	0	0	0	0	24	24
Sum	52	34	36	63	102	100	35	33	30	24	509

**Table 3:** The regression analysis of the estimated fog density values and the RVR values. The coefficients and several related statistical indicators of fitting Equation (3).

Method	$C_{0}$	α	$R^{2}$	*p*-Value	$\varepsilon_{ave}$	$\varepsilon_{max}$
FADE	4.5759	−0.000047	0.2884	0.0000	0.1266	0.8362
JSVC	0.4123	−0.000720	0.2546	0.0002	0.0387	0.1189
SFDE	1.0105	0.0000008	0.0029	0.0455	0.0056	0.0302
JdEg	2.3462	−0.010356	0.8044	0.0000	0.0786	0.3077
GDEn	6.7371	−0.000126	0.3460	0.0000	0.6634	1.0841

$\varepsilon_{ave}$ is the average error; $\varepsilon_{max}$ is the maximum absolutely error.

## Data Availability

No new data were created.
